# Adolescent idiopathic scoliosis (AIS): new gene, new variants

**DOI:** 10.1186/1748-7161-10-S1-O1

**Published:** 2015-01-19

**Authors:** Florina Moldovan, Amani Hassan, Charlotte Zaouter, Soraya Barchi, Patrick Edery, Pierre Drapeau, Stefan Parent, Shunmoogum A Patten

**Affiliations:** 1 Research Center, Sainte-Justine University Hospital Center, Montréal, Canada; 2 Department of Stomatology, Faculty of Dental Medicine, Université de Montréal, Canada; 3 Hospices Civils de Lyon, Lyon, France; Inserm U1028, CNRS UMR5292, University Lyon, France; 4 CRCHUM and Department of Neuroscience, Université de Montréal, Canada; 5 Department of Surgery, Faculty of Medicine, Université de Montréal, Canada

## Introduction

Many chromosomic locations have been linked with the AIS, however, no causative genes have been clearly identified. We recently identified two regions (3q12.1 and 5q13.3) containing possibly causative gene(S) of AIS (Edery et al. 2011). Our recent work identified disease-causing variants in a gene (that we called PFK2) in French AIS families (Patten et al. submitted 2014). We sought to expand on this study and to investigate for novel and rare variants in PFK2 in French Canadian AIS families.

## Methods

32 French Canadian families from Quebec were investigated. The inclusion criteria were Family history of at least 3 members with progressive or non progressive AIS, and on the basis of radiological data including as a phenotype the spinal rotation. DNA was extracted from peripheral blood or saliva. The entire gene was investigated using the classical PCR amplified Sanger Sequencing. All the coding-regions of the gene were amplified, co-segregation analysis was performed and frequency of variants was compared controls of 1000 Genomes. In addition, the pathogenicity of PFK2 mutations was evaluated through *in vivo* functional studies in zebrafish, including the knockdown morpholino approach and gain of function were performed. Wild-type and mutated zebrafish phenotype was assessed morphologically, histologically, by whole-mount *in situ* hybridizations and by microCT (three-dimensional Imaging and Reconstruction of Zebrafish Bone).

## Results

Variants in PFK2 gene were identified in 4 out of 32 Families. Co-segregation analysis revealed these variants in affected members of the same families but not in non-affected members. We identified 4 new missense variants located on the coding regions of PFK2 (3 variants in Exome 11 and 1 in Exome 12) (all absent from the genome variants databases), two rare missense variants (Exome10) (allelic frequency of ~0.6% in the 1000 Genomes), and several common SNPs (missense or intronic, without predicted consequence on amino acid changes). Functional analyses of those variants in zebrafish demonstrated spinal deformity similar to that observed in patients including significant spinal rotation, and bone delayed mineralisation. Interestingly, PFK2 is highly expressed in zebrafish midbrain during the early development, suggesting that AIS may primarily originate from a brain-associated dysfunction.

## Conclusion

Altogether, we identified disease-causing variants in PFK2 in French families and cases and we confirmed the association of novel and rare variants in PFK2 with AIS in French Canadian families.

